# Facial analysis using a new clinical device: The Kattan Facio-meter

**DOI:** 10.4317/jced.55394

**Published:** 2019-01-01

**Authors:** Ehab S. El Kattan, Omnia A. Elhiny, Mohamed E. El Kattan, Aya E. El Kattan, Amira Elsheikh

**Affiliations:** 1 Professor, Department of Orthodontics, Cairo University; 2 Associate Professor, Department of Orthodontics and Pediatric Dentistry, National research Centre; 3 Lecturer, Department of Pediatric Dentistry, Cairo University; 4 Assistant lecturer, Department of Orthodontics and Pediatric Dentistry, National Research Centre; 5 Assistant lecturer, Private Practice

## Abstract

**Background:**

The aim of the study was to attempt to set average faciometric standards for Egyptians using the Kattan Facio-meter.

**Material and Methods:**

The sample consisted of 180 faculty students with age range 17-25 years. It was divided into three groups; Angle Class I, II and III. Class II was further subdivided into divisions 1 and 2. Linear and angular facial measurements in relation to K plane were taken using the Kattan facio-meter. The measurements were correlated to Angle’s classification and between genders.

**Results:**

On comparing the different classes, Class II division 1 showed the statistically highest mean value for Orbitale-soft tissue A; *p*=0.042, Class II divisions 1 and 2 for Orbitale- Labrale superius; *p*=0.002 and soft tissue ANB; *p*<0.001. Females showed significantly higher mean value than males for the upper incisor/K plane; *p*=0.031. Males showed significantly higher mean value for the inter-incisal angle than females; *p*=0.001.

**Conclusions:**

Within the limitations of the current study, it was found that both linear and angular soft tissue measurements conformed to the antroposterior skeletal relation of the jaws and that Class II division 1 was due to protruded maxilla. Males had more prominent lips and deeper mentolabial sulcus. Egyptians had less prominent noses than Caucasians. The Kattan Facio-meter was a valuable tool for clinical analysis without the hazards of irradiation.

** Key words:**Kattan Facio-meter, Angle Class I, II and III.

## Introduction

One of the most important components of orthodontic diagnosis and treatment planning is the soft tissue evaluation. The esthetic concerns of patients are often paramount, which places a greater responsibility on the orthodontist to plan the treatment within the patient’s limits of soft tissue adaptation and contours (1,2). This soft tissue paradigm in diagnosis and treatment planning has emphasized the importance of the clinical examination of soft tissue esthetics as they are the main determinant of the orthodontic treatment limitations (1,3).

Plooij *et al.* concluded that accurate soft tissue analysis doesn’t require the presence of hard tissue data (4). Nevertheless, the absence of stable, accurate references resulted in the fact that soft tissues could not be accurately quantified by laser scanning, or 3D photogrammetry (2,5,6).

To provide an accurate analysis of patients with malocclusion, Lundstrom *et al.* recommended the use of an extra-cranial reference line and the natural head orientation as a substitute or a supplement to intra-cranial lines (7). The K plane (Kattan plane) was suggested for it combined both the anatomical points and the natural head position.8 It extends from SN to SAE bilaterally and was reported to be a reliable and reproducible clinical substitute to the Frankfort horizontal plane (8,9).

Based on the K plane, the Kattan Facio-meter was designed and validated. It was found that it can be used as a reliable 3D non-radiographic extra-oral and intra-oral diagnostic tool that averts patient irradiation and at the same time overcomes the errors produced by conventional methods. It has the ability to make clinical facial measurements in all 3 planes without tilting that could result in the creation of an inclination angle producing errors (10-12).

Consequently, it was of great value to attempt to set average faciometric standards for Egyptians using the Kattan Facio-meter.

## Material and Methods

-Material

The subjects were selected from the students of the Faculty of Oral and Dental medicine, Cairo University. The sample size was 180 subjects based on sample size calculation with 95% confidence interval and 80% power. The age range was 17-25 years (mean 21 years). Before the commencement of the investigation each subject signed an informed consent.

Inclusion criteria

- The subjects were Egyptian in origin; at least both parents were Egyptian.

- They had a full set of permanent teeth.

- No previous orthodontic or orthognathic surgery.

- Free of syndromes and anomalies.

According to Angle’s classification of the molar relationship, the sample was divided into 3 groups.

Group 1 comprised 100 subjects, having molar Class I, straight teeth or minimal crowding 3-4 mm. Group 2 included 50 subjects with molar Class II malocclusion and was further divided into two subgroups: Group A consisted of Class II division 1 subjects and Group B consisted of Class II division 2 subjects. There were 30 subjects with molar Class III in Group 3.

**Figure 1:**
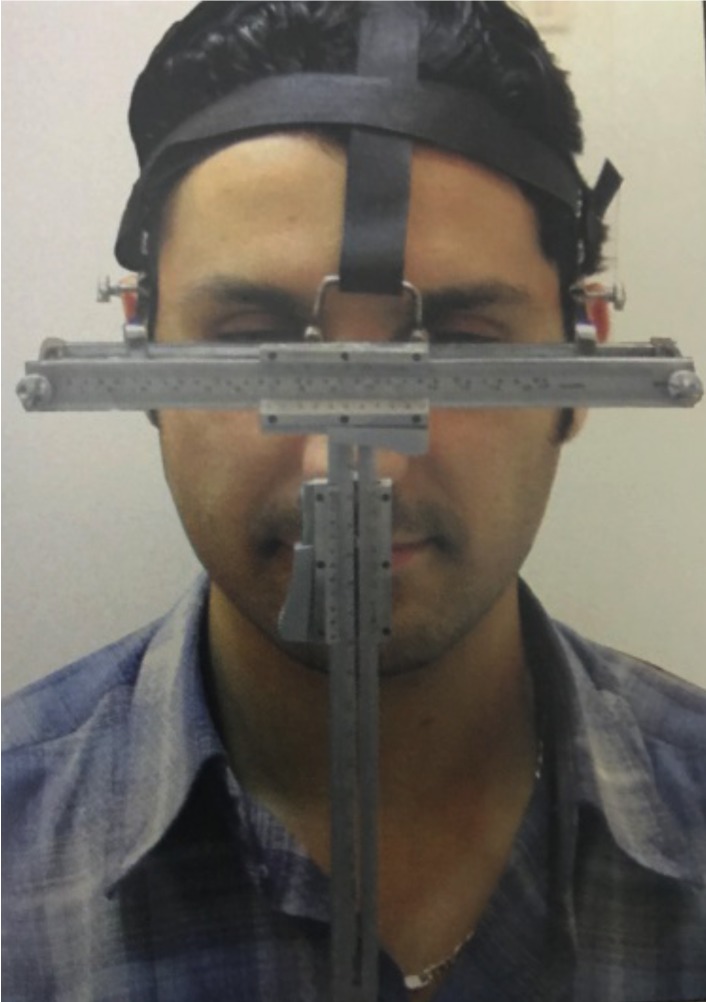
The Kattan facio-meter, frontal view.

**Figure 2:**
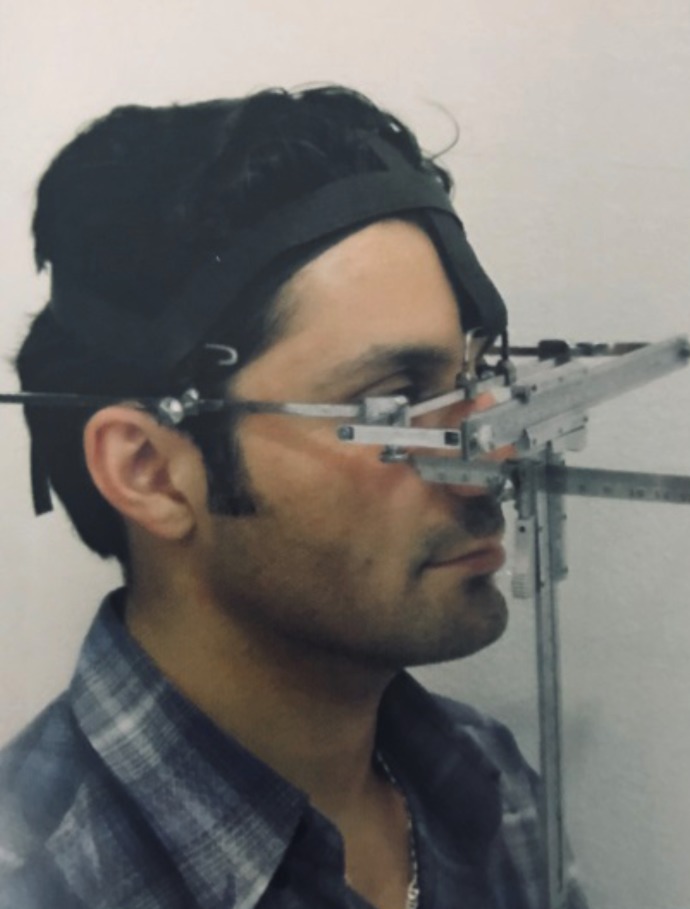
The Kattan facio-meter, 45 degrees view.

The measurement method using the Kattan Facio-meter:

Linear and angular measurements were taken using the Kattan Facio-meter*; designed by El Kattan E. It consisted of an anterior, gradated horizontal bar (x-plane) that rested on soft tissue Nasion in front of the pupils of the eyes and two lateral arms attached to the anterior bar, resting on the superior attachment of the ear. They could be adjusted to fit the lateral dimensions of different faces. It also consists of a gradated, sliding vertical ruler perpendicular to the horizontal bar (y-plane) attached to it a sliding ruler in the z-plane. The appliance was secured in place by bands above and around the back of the head (Figs. 1,2).

The Kattan Facio-meter rested on the soft tissue Nasion of the subjects and was adjusted to the proper facial width. The soft tissue Nasion was identified by a black pencil between the bridge of the nose and the forehead when the subject looked forward at a distant point in the same level of the eyes (10-12)

**Figure 3:**
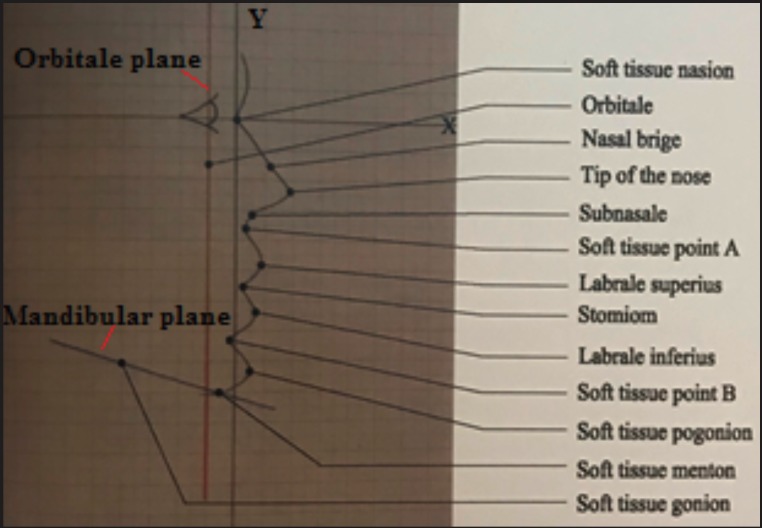
Soft tissue profile drawn on a graph paper.

Soft tissue and dental points were determined, using the Kattan Facio-meter, in the x, y and z dimensions and marked on a graph paper. The profile was drawn, then, for each subject (Fig. 3) and linear and angular measurements were carried out.

The linear measurements were made in relation to Orbitale vertical plane which is a perpendicular line from Orbitale to the K plane. They were as follows: Orbitale-soft tissue Nasion, Orbitale- Nasal bridge, Orbitale- tip of the nose, Orbitale-Subnasale, Orbitale-soft tissue A,

Orbitale- Labrale superius, Orbitale- Stomium, Orbitale-Labrale inferius, Orbitale-Mentolabial sulcus, Orbitale-soft tissue B, Orbitale-soft tissue Pogonion, and Orbitale-soft tissue Menton.

The angular measurements were; soft tissue ANB, inter-incisal angle, lower incisor/mandibular plane, upper incisor/K plane, lower incisor/K plane, and the mandibular plane(Go-Me)/K plane angle.

Statistical analysis was performed with SPSS 15.0® (Statistical Package for Scientific studies) for windows. Means and standard deviations were calculated, ANOVA test was used to compare between the means. Duncan’s post-hoc test was used for pair-wise comparisons between means when the ANOVA test was significant. Student’s t-test was used to compare between the mean measurements of male and female subjects. The significance level of all tests was set at *P*≤0.05.

## Results

-On comparing the means and standard deviations, in addition to the results of ANOVA and Duncan’s tests in the different classes for both the linear and angular measurements, the results were as follows:

The linear measurements in the different classes:

Class II division 1 showed the statistically significant highest mean value for Orbitale-soft tissue A; *p*=0.042, and Class II divisions 1 and 2 for Orbitale- Labrale superius; *p*=0.002. While Class III showed the statistically significant highest mean value for Orbitale-Labrale inferius; *p*=0.001, Orbitale-Mentolabial sulcus (*p*=0.035) and Orbitale-soft tissue Pogonion (*p*=0.003). Class I showed the statistically significant highest mean value for Orbitale-soft tissue B; *p*=0.013.

There was no significant difference for the other linear measurements between classes.

The angular measurements in the different classes:

Classes II division 1 and division 2 showed the statistically significant highest mean value for soft tissue ANB; *p*<0.001, and Class II division 1 for lower incisor/mandibular plane; *p*=0.008. There was no significant difference for the other angular measurements between classes.

-When the males and females’ means and standard deviations, in addition to the results of ANOVA and Duncan’s tests were compared in the different classes the results were as follows:

Class I linear measurements:

The mean value of the measurements Orbitale- tip of the nose (*p*=0.043), Orbitale-Labrale superius (*p*=0.023) and Orbitale-Labrale inferius (*p*=0.030) were significantly higher in males than females. While females showed significantly higher mean values than males for Orbitale-Mentolabial sulcus; *p*=0.038.

There was no significant difference for the other linear measurements between males and females.

Class I angular measurements:

Males showed a significantly higher mean value than females as regards Go-Me/K plane angle, and the inter-incisal angle; *p*=0.04 and *p*=0.001, respectively. While females showed significantly higher mean value than males for the upper incisor/K plane; *p*=0.031. There was no significant difference for the other angular measurements between males and females.

Classes II division 1 and 2 measurements:

In Class II division 1 linear measurements, males showed a statistically significant higher mean value for Orbitale-soft tissue Nasion; *p*=0.030, Orbitale-Nasal bridge; *p*=0.027, Orbitale-Labrale superius and labrale inferius; *p*=0.011 and *p*=0.029, respectively. As for the angular measurements, the mean value of upper central-K plane was significantly higher in females; *p*=0.009, while the mean of Mandible-K plane, lower central-K plane and lower central-upper central was significantly higher in males; *p*<0.001, *p*=0.004 and *p*=0.026, respectively.

In Class II division 2 linear measurements, the mean value of Orbitale-Labrale superius and inferius was significantly higher in males; *p*=0.001 and *p*=0.017, respectively. While Orbitale-Mentolabial sulcus was significantly higher in females, *p*=0.023. As for the angular measurements, the mean value of lower central-upper central was significantly higher in males, p=0.014 and upper central-K plane was significantly higher in males, *p*=0.031.

Class III measurements:

Descriptive statistics including means and standard deviations, in addition to the results of ANOVA and Duncan’s tests for comparing males and females in Class III were shown in  Table 1 and  Table 2 for the linear and angular measurements, respectively.

**Table 1:**
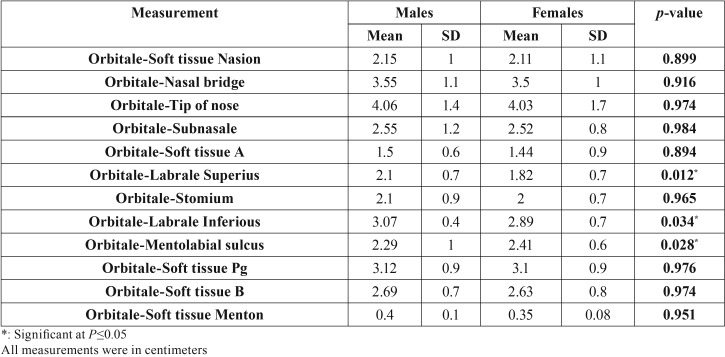
Linear measurements in Class III; males and females.

**Table 2:**
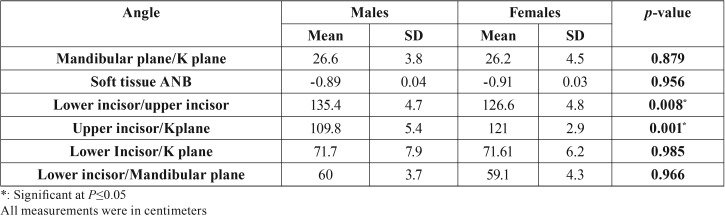
Angular measurements in Class III; males and females.

## Discussion

In orthodontic treatment, the physiologic limits are narrower than the anatomic limits and the tolerance for soft tissue adaptation is by far less. Soft tissue analysis in the natural head position is an important step in orthodontic decision making (3).

Henceforth, it was important to set average faciometric standards for Egyptians using the Kattan Facio-meter. To the best of our knowledge, it could be considered the only reliable and validated device available to make clinical soft tissue measurements, in the natural head position, without the previously reported hazards of irradiation or the errors of other conventional methods (10-14). It has the ability to measure the facial outlines in three dimensions; vertically, horizontally and antro-posteriorly without tilting that might create an angle of inclination producing measurements’ errors (11,12). The Facio-meter’s concept depends on the K plane which was shown by El Kattan *et al.* to be a reliable clinical substitute to Frankfurt horizontal and acts as the true horizontal of the head (8,9).

Bishara *et al.* in 1990 concluded that the Egyptian population was comparatively homogenous; hence the collected sample could be considered as a representative of the total population (15).

Linear and angular measurements were taken and compared between classes and males and females. Linear measurements were taken from the Orbitale vertical plane which was a perpendicular line from Orbitale to the K plane. This was a novel method of recording the measurements. This method was used since soft tissue Orbitale was considered a reliable point when determined clinically using the facio-meter.

The soft tissue ANB angle can vary with the length of the cranial base and/or rotation of the jaws; a finding that forced different authors to use both linear and angular measurements to determine the accurate antro-posterior positions of the jaws (16-22). That was why Orbitale-soft tissue A in addition to soft tissue ANB angle was measured.

The evaluation of the measurement “Orbitale-soft tissue A” showed that Class II divisions 1 and 2 had the significantly highest mean value, followed by Classes II and III, respectively. This revealed that in the Egyptian population, Class II was mainly due to the forward position of the maxilla; compared to the Japanese population where Ishii *et al.* suggested that it was due to backward rotation of the mandible. However, they evaluated the mandibular position by measuring the horizontal distance Sella-Pogonion (23), compared to Orbitale-soft tissue Pogonion and soft tissue B, used in this study, where Class III showed the significantly highest mean value followed by Class I then Class II. This finding suggested that Class III was mainly due to mandibular forward position.

The measurements Orbitale-Labrale superius and labrale inferius suggested that males exhibited greater lip protrusion than females. This was in agreement with other studies on American Negros and Egyptian children, but not among Caucasians (24,25). This finding also explains the significantly deeper mentolabial sulcus results found in males. Munir *et al.* reported similar outcomes and suggested that it might have been due to thicker lower lips in males (26).

Compared to Caucasians investigated in other studies, Egyptians had generally less prominent noses and males’ noses were more prominent than females when the linear measurement Orbitale-tip of the nose was considered, however the studies on Caucasians used the angle of nasal convexity (26,27).

When the angular measurements were investigated, it was found that Class II divisions 1 and 2 showed the statistically significant highest mean value regarding soft tissue ANB; followed by Classes I and III, respectively. This affirmed the results of the linear measurement Orbitale-soft tissue A and conformed with other studies (28) and revealed that the soft tissue measurements were strongly correlated to the underlying antro-posterior position of the maxilla and mandible as previously reported (4).

Similarly, the dental measurement “lower incisor/mandibular plane angle” showed a greater degree of incisor proclination in Class II division 1 which could be due to the dental compensation resulting from the presence of proclined upper incisors (29,30). The interincisal angle in Class I was found to be more acute in Egyptians than Caucasians, this was in accordance with previous studies on Egyptians (31,32). Furthermore, it was significasntly more acute in females than males which conformed with Fahmy suggesting that Egyptian females had more dental protrusion (32).

Eventually, it was deduced that the Kattan Facio-meter was valuable for clinical intra-oral and extra-oral, linear and angular measurements. In the future, it could be easily digitized and connected to a computer to facilitate the measuring process and allow drawing of the profile outline. This is important in the diagnosis and treatment planning of orthodontic cases, orthognathic cases and functional analysis.

## Conclusions

Within the limitations of the current study, it was found that:

- In Class II division 1 the maxilla was mainly protruded, while in Class III the mandible was in a forward position.

- Males had more prominent lips and deeper mentolabial sulcus and females had more dental protrusion.

- Egyptians had less prominent noses than Caucasians and the prominence was more observed in males.

- Linear and angular soft tissue measurements conformed to the antroposterior skeletal relation of the jaws.

- The Kattan Facio-meter was a valuable tool for clinical analysis without the hazards of irradiation.

- Future digitization could be easily done to the Kattan Facio-meter and connecting it to the computer for performing various orthodontic and orthognathic analyses.
